# Apolipoprotein E and sex modulate fatty acid metabolism in a prospective observational study of cognitive decline

**DOI:** 10.1186/s13195-021-00948-8

**Published:** 2022-01-03

**Authors:** Raúl González-Domínguez, Pol Castellano-Escuder, Sophie Lefèvre-Arbogast, Dorrain Y. Low, Andrea Du Preez, Silvie R. Ruigrok, Hyunah Lee, Catherine Helmer, Mercè Pallàs, Mireia Urpi-Sarda, Alex Sánchez-Pla, Aniko Korosi, Paul J. Lucassen, Ludwig Aigner, Claudine Manach, Sandrine Thuret, Cécilia Samieri, Cristina Andres-Lacueva

**Affiliations:** 1 Biomarkers and Nutrimetabolomics Laboratory, Food Innovation Network (XIA), Nutrition and Food Safety Research Institute (INSA), Faculty of Pharmacy and Food Sciences, University of Barcelona, Av. de Joan XXIII, 27-31, 08028 Barcelona, Spain; 2 CIBER Fragilidad y Envejecimiento Saludable (CIBERfes), Instituto de Salud Carlos III, 28029 Madrid, Spain; 3 Department of Genetics, Microbiology and Statistics, University of Barcelona, 08028 Barcelona, Spain; 4 Inserm, Bordeaux Population Health Research Center, UMR 1219, University of Bordeaux, F-33000 Bordeaux, France; 5 Université Clermont Auvergne, INRAE, UNH, F-63000 Clermont Ferrand, France; 6 Department of Basic and Clinical Neuroscience, Maurice Wohl Clinical Neuroscience Institute, Institute of Psychiatry, Psychology and Neuroscience, King’s College London, London, SE5 9NU UK; 7 Brain Plasticity Group, Swammerdam Institute for Life Sciences, Center for Neuroscience, University of Amsterdam, 1098 XH Amsterdam, The Netherlands; 8 Pharmacology Section, Department of Pharmacology, Toxicology and Medicinal Chemistry, Faculty of Pharmacy and Food Sciences, and Institut de Neurociències, University of Barcelona, 08028 Barcelona, Spain; 9 Institute of Molecular Regenerative Medicine, Spinal Cord Injury and Tissue Regeneration Center Salzburg, Paracelsus Medical University, 5020 Salzburg, Austria

**Keywords:** Cognitive decline, Fatty acids, Apolipoprotein E, Sex, Acyl-carnitines, Metabolomics

## Abstract

**Background:**

Fatty acids play prominent roles in brain function as they participate in structural, metabolic and signaling processes. The homeostasis of fatty acids and related pathways is known to be impaired in cognitive decline and dementia, but the relationship between these metabolic disturbances and common risk factors, namely the ɛ4 allele of the apolipoprotein E (ApoE-ɛ4) gene and sex, remains elusive.

**Methods:**

In order to investigate early alterations associated with cognitive decline in the fatty acid-related serum metabolome, we here applied targeted metabolomics analysis on a nested case-control study (N=368), part of a prospective population cohort on dementia.

**Results:**

When considering the entire study population, circulating levels of free fatty acids, acyl-carnitines and pantothenic acid were found to be increased among those participants who had greater odds of cognitive decline over a 12-year follow-up. Interestingly, stratified analyses indicated that these metabolomic alterations were specific for ApoE-ɛ4 non-carriers and women.

**Conclusions:**

Altogether, our results highlight that the regulation of fatty acids and related metabolic pathways during ageing and cognitive decline depends on complex inter-relationships between the ApoE-ε4 genotype and sex. A better understanding of the ApoE-ɛ4 and sex dependent modulation of metabolism is essential to elucidate the individual variability in the onset of cognitive decline, which would help develop personalized therapeutic approaches.

**Supplementary Information:**

The online version contains supplementary material available at 10.1186/s13195-021-00948-8.

## Background

Cognitive decline (CD), Alzheimer’s disease (AD) and other forms of dementia are major health concerns due to the population aging worldwide. While the pathological processes that underlie these disorders are thought to develop for decades before clinical symptoms occur, their molecular and cellular mechanisms remain poorly understood. Recent evidence suggests that aberrant lipid metabolism represents a cornerstone in CD pathogenesis, involving disturbances in the homeostasis of cholesterol, phospholipids, sphingolipids and other lipid classes, both in the brain and at systemic levels [1]. Among lipids, fatty acids are recognized to play important roles in neurotransmission, neuronal membrane structure and plasticity, neuroinflammation, and many other processes. Furthermore, free fatty acids (FFAs) participate in energy metabolism via mitochondrial β-oxidation [1]. In this respect, numerous studies have suggested that impaired energy metabolism, characterized by reduced glucose utilization and mitochondrial dysfunction, could be another primary hallmark of neurodegeneration [2]. Altogether, fatty acid metabolism and related metabolic pathways appear to be essential determinants in the onset of CD.

The ɛ4 allele of the apolipoprotein E (ApoE) gene and female sex are strong risk factors for CD and AD [3]. ApoE has a paramount role in lipid homeostasis by regulating the transport and metabolism of cholesterol, triglycerides and phospholipids in multiple tissues. In particular, CD subjects carrying the ApoE-ɛ4 allele suffer from early and sharpened glucose hypometabolism, which is normally accompanied by enhanced fatty acid metabolism [4]. Furthermore, it has been reported that the ApoE genotype is associated with AD and related biomarkers in a sex-dependent manner, with the ɛ4 allele conferring greater risk for women [5, 6]. In particular, Neu et al. reported that, among ApoE-ɛ4 carriers, the risk for developing AD or mild cognitive impairment (MCI) is not significantly different between men and women when considering a broad age window (55-85 y), but women are at increased risk compared to men at younger ages (65-75 y for AD, 55-70 y for MCI) [7]. In this context, metabolomics has demonstrated great potential to investigate the profound impact of CD and dementia on metabolism and the final phenotype [8, 9]. However, in most of these previously published studies, the ApoE genotype and sex were not considered at all, or were merely used as covariates for adjusting statistical models, which may have masked the discovery of specific associations between CD/AD and metabolite levels depending on these risk factors. Accordingly, stratified analyses are mandatory for elucidating the mechanisms underlying the intertwined modulation of CD by ApoE-ɛ4 and sex, as recently proposed by others as well [10–12].

In this study, we explored the role of the ApoE-ɛ4 carrier status and sex in modulating fatty acid metabolism and related pathways in early CD, before the appearance of dementia symptoms. To this end, a targeted metabolomics approach was applied to serum samples from a prospective cohort of older subjects from the Three-City (3C) Cohort [13].

## Methods

### Study design

The study population consisted of selected participants of the 3C study, a prospective multi-center population-based cohort on dementia that comprises older persons aged above 65 years at inclusion in 1999 [13]. At baseline, sociodemographic and lifestyle characteristics, medical information, cognitive testing, anthropometric measurements, and fasting blood samples were gathered from all participants. Then, visits were scheduled every 2-3 years during a 12-year follow-up for neuropsychological assessment that included five neuropsychological tests, namely the Mini-Mental State Examination test (MMSE), Benton Visual Retention Test (BVRT), Isaac’s Set Test (IST), Trail-Making Test part A (TMTA) and Trail-Making Test part B (TMTB) [14–17]. The ApoE-ε4 genotype was defined dichotomously as carrying at least one ε4 allele (ε4+) versus the absence of ε4 allele (ε4-), using the genetic testing method detailed previously [18]. Fasting plasma levels of glucose, creatinine, cholesterol (total, LDL-C, HDL-C), and triglycerides were measured at baseline by routine enzymatic methods [13].

In particular, the present study leveraged a case-control sub-sample on CD among participants from the center of Bordeaux, which was built in a previous study aimed to identify diet-related metabolites associated with CD [19]. Briefly, eligible participants were selected among those who were not diagnosed with dementia and had available serum samples at baseline, and had at least one repeated cognitive evaluation over the 12-year follow-up. Individual slopes of cognitive change were then evaluated by applying linear mixed models, using a composite score of global cognition as the primary outcome, which was defined at each follow-up visit as the average of Z-scores of the five neuropsychological tests mentioned above (MMSE, BVRT, IST, TMTA, TMTB). Accordingly, cases were defined as the participants with the worst slopes of CD, who were matched to a control (i.e., a participant with a slope of CD better than the population median) with the same age, gender and education level [19]. From this original sample set, we here focused on the participants with available ApoE-ε4 genotyping data (N=368). The study was performed in accordance with the principles contained in the Declaration of Helsinki. The Consultative Committee for the Protection of Persons participating in Biomedical Research at Kremlin-Bicêtre University Hospital (Paris, France) approved the 3C study protocol, and all participants provided written consent.

### Metabolomics analysis of serum samples

Serum samples were analyzed using a targeted metabolomics platform for the simultaneous quantitation of 55 metabolites involved in fatty acid metabolism and related metabolic pathways, including 17 free fatty acids (FFAs), 17 acyl-carnitines (ACs), 12 energy-related metabolites and 9 B-group vitamins (Table S1). To this end, serum samples were subjected to protein precipitation [19], and analyses were then carried out by ultra-high performance liquid chromatography coupled to tandem mass spectrometry, as described elsewhere [20].

### Statistical analysis

The metabolomics data matrix was first pre-processed to remove metabolites with more than 20% missing values in all the study groups, and the remaining missing values were imputed using the kNN algorithm (k = 10). Data normality was checked by inspecting probability plots. Afterwards, data were log transformed and Pareto scaled, and then subjected to statistical analysis using the R/Bioconductor POMA package. Linear models were built using the *limma* package to look for metabolite differences between the study groups, either by considering the whole study population (i.e., CD vs. CTL) as well as after stratifying according to the ApoE-ε4 genotype (i.e., CD_ε4+_, CD_ε4-_, CTL_ε4+_, CTL_ε4-_) and sex (i.e., CD_F_, CD_M_, CTL_F_, CTL_M_). In addition to the matching variables (i.e., sex, age and education level), the ApoE-ε4 genotype and BMI were also used as covariates for model adjustment, except for the ApoE-stratified and sex-stratified analyses, where the stratification variables ApoE-ε4 and sex were excluded as covariates, respectively. Furthermore, Pearson’s correlations were computed between metabolomics, biochemical (i.e., glucose, creatinine, cholesterol, triglycerides) and neuropsychological (i.e., MMSE, BVRT, IST, TMTA, TMTB) data within groups stratified according to the ApoE-ε4 genotype and sex (i.e., F_ε4+_, F_ε4-_, M_ε4+_, M_ε4-_). All the statistical analyses were adjusted for multiple comparisons using the Benjamini-Hochberg false discovery rate (FDR). FDR-corrected p-values below 0.1 were considered as statistically significant.

## Results

### Characteristics of the study population

The participants (N=368) were on average 75 years-old at baseline, 66% were female, and approximately 30% had secondary school education or higher (Table 1). Compared to controls, the participants with the worst slopes of CD over the 12-year follow-up were twice more often carriers of the ApoE-ε4 allele (see Table S2 for further details about the ApoE stratification). In this respect, the frequency of the ε4+ genotype was similar between males and females within CD cases, and slightly higher among females within the control group. As expected, medication intake was significantly higher within cases, whereas lower scores for the five neuropsychological tests employed here (i.e., MMSE, BVRT, IST, TMTA and TMTB) were observed among CD participants. These scores remained stable within the control group over the 12 years, whereas cases suffered a considerable decline at the study endpoint (Table S3). At the end of the 12-year follow-up, 106 individuals were diagnosed with dementia among cases, whereas only 7 control subjects developed dementia symptoms.

**Table 1:** Baseline demographic and clinical characteristics of the study participants. For continuous variables, results are expressed as mean ± SD

	Cases			Controls		
	All	Female	Male	All	Female	Male
N	202	133	69	166	110	56
Age (years)	75.8 ± 4.4	76.1 ± 4.4	75.2 ± 4.5	75.1 ± 4.1	75.5 ± 4.0	74.4 ± 4.2
Sex (% female)	65.8	-	-	66.3	-	-
Education level, ≥ secondary school (%)	29.7	30.1	29.0	31.9	31.8	32.1
ApoE-ε4+ frequency (%)	25.7	25.6	26.1	12.0	13.6	8.9
BMI (kg m^-2^)	26.8 ± 4.3	26.4 ± 4.6	27.6 ± 3.7	26.3 ± 3.7	26.1 ± 4.2	26.7 ± 2.6
Number of medications regularly consumed	4.9 ± 2.8	5.2 ± 2.7	4.2 ± 2.7	3.9 ± 2.3	4.1 ± 2.1	3.4 ± 2.7
MMSE (points)	27.0 ± 2.2	27.0 ± 2.2	26.9 ± 2.2	27.9 ± 1.7	27.8 ± 1.8	28.2 ± 1.4
BVRT (points)	10.8 ± 2.1	10.6 ± 2.1	11.1 ± 2.1	11.8 ± 1.9	11.6 ± 2.0	12.2 ± 1.7
IST (points)	27.8 ± 5.8	27.7 ± 5.7	28.0 ± 6.4	31.2 ± 6.1	31.2 ± 6.4	31.1 ± 5.7
TMTA (number of correct displacements per minute)	24.1 ± 8.0	23.5 ± 7.7	25.4 ± 8.5	29.3 ± 9.3	29.1 ± 9.3	29.7 ± 9.4
TMTB (number of correct displacements per minute)	10.0 ± 5.8	9.8 ± 5.5	10.6 ± 6.5	14.5 ± 6.7	14.4 ± 6.7	14.8 ± 6.6
Glucose (mmol L^-1^)	5.5 ± 1.6	5.4 ± 1.8	5.5 ± 1.3	5.1 ± 1.0	4.9 ± 0.7	5.6 ± 1.4
Creatinine (mmol L^-1^)	83.1 ± 22.5	75.9 ± 14.2	97.0 ± 28.3	79.3 ± 18.1	73.6 ± 14.9	90.7 ± 18.5
Cholesterol (mmol L^-1^)	5.8 ± 0.9	5.9 ± 0.9	5.6 ± 0.9	5.8 ± 1.0	5.9 ± 1.0	5.7 ± 0.9
LDL-C (mmol L^-1^)	3.6 ± 0.8	3.7 ± 0.8	3.6 ± 0.8	3.6 ± 0.9	3.7 ± 1.0	3.6 ± 0.7
HDL-C (mmol L^-1^)	1.6 ± 0.4	1.7 ± 0.4	1.4 ± 0.4	1.6 ± 0.4	1.6 ± 0.4	1.4 ± 0.3
Triglycerides (mmol L^-1^)	1.4 ± 0.8	1.3 ± 0.7	1.4 ± 0.9	1.3 ± 0.7	1.2 ± 0.5	1.4 ± 0.9

Abbreviations: ApoE-ε4+, carrier of the ε4 allele of the apolipoprotein E gene; BMI, body mass index; MMSE, Mini-Mental State Examination test; BVRT, Benton Visual Retention Test; IST, Isaac’s Set Test; TMTA, Trail-Making Test part A; TMTB, Trail-Making Test part B; LDL-C, low-density lipoprotein cholesterol; HDL-C, high-density lipoprotein cholesterol.

Furthermore, we also considered the possible confounding effect of diet and the consumption of glucose lowering drugs. The analysis of the dietary data acquired through food frequency questionnaires evidenced that the study groups showed similar frequency of consumption of the main food groups that can affect the circulating levels of fatty acids and related metabolites (e.g., fish, meat, dairy products) [19], so the influence of diet on our metabolomics results can be assumed to be negligible. On the other hand, a borderline significant difference was observed in the consumption of glucose lowering drugs between cases and controls (p = 0.039). This was expected as the prevalence of diabetes was considerably higher within cases (i.e., 12.9% among cases, 5.7% among controls), in line with previous studies reporting that diabetes is a well-known risk factor for cognitive decline [21]. However, no differences were observed according to the ApoE genotype or sex.

### Impact of ApoE-ε4 genotype and sex on circulating levels of fatty acid-related metabolites in a prospective cohort on CD

The participants with greater odds of CD over the 12-year follow-up had increased serum levels of FFAs, ACs and pantothenic acid at baseline compared to controls when considering the entire study population, as shown in Table 2 (Table S4 lists the metabolite concentrations within each study group; Table S5 lists the metabolite concentrations within ApoE-stratified subgroups). Interestingly, individuals taking glucose lowering drugs (i.e., patients with diabetes and related disorders) also had significantly higher serum concentrations of free fatty acids (palmitic acid, margaric acid, oleic acid, linoleic acid, docosatetraenoic acid) and acetyl-carnitine, in line with previous studies [22]. This could suggest that case-control differences in the fatty acid-related metabolome could be mediated, at least in part, by the diabetic complications underlying CD. Accordingly, we favored not to adjust the models for the consumption of these drugs as additional covariates, since adjusting for a mediator may delete an association.

**Table 2:** Fatty acid-related metabolites identified by lineal modeling to be associated with cognitive decline, ApoE-ε4 genotype and sex

	Whole study population	ApoE-ε4 stratification				Sex stratification			
	CD *vs.* CTL	CD_ε4+_ *vs.* CTL_ε4+_	CD_ε4-_ *vs.* CTL_ε4-_	CTL_ε4+_ *vs.* CTL_ε4-_	CD_ε4+_ *vs.* CD_ε4-_	CD_F_ *vs.* CTL_F_	CD_M_ *vs.* CTL_M_	CTL_F_ *vs.* CTL_M_	CD_F_ *vs.* CD_M_
*Free fatty acids*									
Myristic acid	1.38 (3.1·10^-2^)	1.05 (NS)	1.39 (6.0·10^-2^)	1.12 (NS)	1.08 (NS)	1.53 (3.3·10^-2^)	1.13 (NS)	1.49 (NS)	2.03 (1.3·10^-2^)
Palmitic acid	1.09 (NS)	0.50 (5.3·10^-2^)	1.27 (NS)	2.22 (3.9·10^-3^)	0.87 (NS)	1.16 (NS)	0.97 (NS)	0.90 (NS)	1.07 (NS)
Palmitoleic acid	1.14 (NS)	0.60 (NS)	1.29 (NS)	2.08 (1.1·10^-2^)	0.96 (NS)	1.22 (NS)	1.00 (NS)	1.25 (NS)	1.51 (6.4·10^-2^)
Margaric acid	1.13 (NS)	0.81 (NS)	1.21 (NS)	1.56 (NS)	1.05 (NS)	1.18 (NS)	1.05 (NS)	0.97 (NS)	1.08 (NS)
Stearic acid	1.03 (NS)	0.39 (5.5·10^-3^)	1.25 (NS)	2.47 (1.7·10^-3^)	0.78 (NS)	1.07 (NS)	0.96 (NS)	0.88 (NS)	0.98 (NS)
Oleic acid	1.19 (NS)	0.50 (7.8·10^-2^)	1.41 (2.2·10^-2^)	2.28 (2.3·10^-3^)	0.80 (NS)	1.26 (NS)	1.06 (NS)	0.99 (NS)	1.18 (NS)
Linoleic acid	1.22 (9.7·10^-2^)	0.78 (NS)	1.25 (2.2·10^-2^)	1.62 (4.4·10^-2^)	0.94 (NS)	1.29 (6.1·10^-2^)	1.10 (NS)	0.86 (NS)	1.01 (NS)
Linolenic acid	1.12 (NS)	0.65 (NS)	1.25 (6.7·10^-2^)	1.74 (9.0·10^-3^)	0.91 (NS)	1.26 (7.8·10^-2^)	0.89 (NS)	0.93 (NS)	1.32 (NS)
Arachidonic acid	1.05 (NS)	0.60 (NS)	1.18 (NS)	1.87 (6.0·10^-3^)	0.95 (NS)	1.15 (NS)	0.89 (NS)	0.78 (8.2·10^-2^)	1.01 (NS)
Eicosapentaenoic acid	0.99 (NS)	0.68 (7.8·10^-2^)	1.07 (NS)	1.43 (3.9·10^-2^)	0.91 (NS)	1.08 (NS)	0.84 (NS)	0.80 (NS)	1.03 (NS)
Docosatetraenoic acid	1.18 (9.7·10^-2^)	0.73 (NS)	1.30 (1.2·10^-2^)	1.76 (1.7·10^-3^)	0.99 (NS)	1.31 (3.3·10^-2^)	0.97 (NS)	0.81 (8.2·10^-2^)	1.10 (NS)
Docosapentaenoic acid	1.26 (NS)	0.75 (NS)	1.39 (3.4·10^-2^)	1.86 (1.7·10^-2^)	1.01 (NS)	1.38 (3.9·10^-2^)	1.04 (NS)	0.80 (NS)	1.06 (NS)
Docosahexaenoic acid	1.09 (NS)	0.60 (7.8·10^-2^)	1.23 (NS)	1.75 (9.0·10^-3^)	0.86 (NS)	1.24 (NS)	0.85 (NS)	0.74 (5.4·10^-2^)	1.07 (NS)
*Acyl-carnitines*									
Acetyl-L-carnitine	1.40 (1.6·10^-2^)	0.88 (NS)	1.54 (4.5·10^-3^)	1.75 (2.0·10^-2^)	1.00 (NS)	1.43 (3.3·10^-2^)	1.35 (NS)	0.96 (NS)	1.02 (NS)
Octanoyl-L-carnitine	1.46 (2.3·10^-2^)	0.87 (NS)	1.62 (1.2·10^-2^)	1.50 (NS)	0.80 (NS)	1.60 (3.3·10^-2^)	1.23 (NS)	0.77 (NS)	1.01 (NS)
Decanoyl-L-carnitine	1.43 (3.2·10^-2^)	0.79 (NS)	1.61 (1.2·10^-2^)	1.51 (NS)	0.73 (NS)	1.54 (3.3·10^-2^)	1.24 (NS)	0.78 (NS)	0.97 (NS)
Undecanoyl-L-carnitine	1.20 (1.6·10^-2^)	0.96 (NS)	1.25 (9.7·10^-3^)	1.16 (NS)	0.89 (NS)	1.23 (3.3·10^-2^)	1.15 (NS)	0.96 (NS)	1.02 (NS)
Lauroyl-L-carnitine	1.44 (1.6·10^-2^)	0.79 (NS)	1.62 (6.0·10^-3^)	1.77 (3.6·10^-2^)	0.87 (NS)	1.56 (3.3·10^-2^)	1.22 (NS)	0.73 (8.2·10^-2^)	0.94 (NS)
Myristoyl-L-carnitine	1.34 (1.6·10^-2^)	0.93 (NS)	1.44 (6.0·10^-3^)	1.62 (1.2·10^-2^)	1.04 (NS)	1.39 (3.3·10^-2^)	1.24 (NS)	0.89 (NS)	1.00 (NS)
Palmitoyl-L-carnitine	1.27 (2.2·10^-2^)	1.05 (NS)	1.32 (1.2·10^-2^)	1.44 (4.4·10^-2^)	1.14 (NS)	1.32 (3.3·10^-2^)	1.18 (NS)	0.74 (8.2·10^-2^)	0.82 (NS)
Oleoyl-L-carnitine	1.33 (1.8·10^-2^)	0.89 (NS)	1.45 (9.7·10^-3^)	1.58 (8.0·10^-2^)	0.97 (NS)	1.39 (3.3·10^-2^)	1.24 (NS)	0.80 (NS)	0.89 (NS)
Linoleoyl-L-carnitine	1.38 (1.6·10^-2^)	1.11 (NS)	1.43 (9.7·10^-3^)	1.36 (NS)	1.06 (NS)	1.40 (3.3·10^-2^)	1.33 (NS)	0.54 (1.2·10^-3^)	0.57 (1.8·10^-3^)
*Other metabolites*									
Citric acid	0.95 (NS)	0.35 (5.3·10^-2^)	1.16 (NS)	2.80 (3.6·10^-2^)	0.84 (NS)	0.88 (NS)	1.11 (NS)	1.09 (NS)	0.87 (NS)
Oxaloacetic acid	0.97 (NS)	2.88 (NS)	0.78 (NS)	0.41 (7.0·10^-2^)	1.50 (NS)	0.93 (NS)	1.05 (NS)	0.84 (NS)	0.74 (NS)
Creatine	1.00 (NS)	0.59 (NS)	1.11 (NS)	1.40 (NS)	0.74 (NS)	0.95 (NS)	1.11 (NS)	3.30 (1.7·10^-9^)	2.84 (3.8·10^-8^)
Creatinine	1.10 (NS)	1.45 (NS)	1.04 (NS)	0.73 (8.0·10^-2^)	1.02 (NS)	1.03 (NS)	1.26 (NS)	0.61 (4.9·10^-4^)	0.50 (1.8·10^-9^)
Pantothenic acid	1.33 (1.6·10^-2^)	1.34 (NS)	1.32 (1.2·10^-2^)	1.23 (NS)	1.25 (NS)	1.37 (3.3·10^-2^)	1.25 (NS)	1.07 (NS)	1.16 (NS)

The results are expressed as fold changes (i.e., the ratio between the mean concentrations detected in the two study groups being compared), with FDR-corrected p-values in brackets.

Abbreviations: CD, cognitive decline; CTL, control; CD_ε4+_, cognitive decline individuals carrying the ε4 allele of the apolipoprotein E gene; CD_ε4-_, cognitive decline individuals non-carrying the ε4 allele of the apolipoprotein E gene; CTL_ε4+_, control individuals carrying the ε4 allele of the apolipoprotein E gene; CTL_ε4-_, control individuals non-carrying the ε4 allele of the apolipoprotein E gene; CD_F_, cognitive decline female individuals; CD_M_, cognitive decline male individuals; CTL_F_, control female individuals; CTL_M_, control male individuals; NS, non-significant.

After subjects’ stratification according to the ApoE-ε4 genotype, these disturbances were only replicated among the non-carrier population. Conversely, CD cases carrying the ε4 allele showed a decreased content of FFAs and citric acid, whereas no significant changes were found for ACs. In this respect, it should also be noted that the ε4+ genotype was characterized by a richer lipid profile, with increased circulating content of FFAs and ACs, but only within the control subjects. This was in turn accompanied by increased levels of citric acid, and decreased levels of oxaloacetic acid and creatinine. Similarly, sex stratification also supported a differential association between CD and the fatty acid-related metabolome among female and male participants. In line with the results described above, CD was associated with increased FFAs, ACs and pantothenic acid only among females, and similar results were observed for ACs and pantothenic acid within men but without reaching statistical significance. In addition, we also found significant metabolomics differences according to sex, with creatine being more abundant in women, and with a higher content of ACs and creatinine in men. Regarding FFAs, the concentrations of saturated and monounsaturated species were higher in women (significant among CD cases, and the same trend was observed within controls), whereas serum levels of long chain polyunsaturated fatty acids (PUFAs) were increased in men (only within the control participants).

### Association of fatty acid-related metabolites with biochemical and neuropsychological variables

Correlation analyses were conducted to investigate the relationship between serum metabolites, biochemical (i.e., glucose, creatinine, total, LDL and HDL-cholesterol, triglycerides) and neuropsychological (i.e., MMSE, BVRT, IST, TMTA, TMTB) variables. To explore possible differential associations depending on the ApoE-ε4 genotype and sex, the study population was stratified into four groups: female carriers (F_ε4+_, N=49), female non-carriers (F_ε4-_, N=194), male carriers (M_ε4+_, N=23), and male non-carriers (M_ε4-_, N=102) (Figure S1). As shown in Table 3, plasma glucose was consistently associated with various fatty acid-related metabolites among women, regardless of the ApoE genotype, including FFAs, ACs and other energy-related metabolites (e.g., creatinine, lactic acid, pyruvic acid). However, these findings were not observed within male participants, except for some FFAs in the ApoE-ε4+ genotype. Creatinine was also differentially associated with serum levels of FFAs (negative association only among men), ACs (positive association in the four study groups) and pantothenic acid (positive association only among women). Regarding plasmatic lipid parameters, total and LDL cholesterol were positively correlated with FFA levels in all study groups, with the exception of F_ε4-_ participants, who displayed an opposite direction of association. Furthermore, HDL-C and triglycerides showed a consistent positive association with FFAs, whereas the correlation with ACs was significant only within ε4 non-carriers (positive association in women, negative association in men). Among neuropsychological variables, the MMSE score was significantly associated with serum FFAs and ACs only within men, but in opposite directions depending on the ApoE-ε4 genotype (positive association among non-carriers, negative association among carriers). The IST score was associated with FFAs in a sex-dependent manner (positive association among males, negative association among females), whereas the association between FFAs and the TMTA was sex-specific (positive association only among men). On the other hand, serum levels of ACs and FFAs showed an ApoE-dependent correlation with TMTA and TMTB, respectively, but only among female subjects (positive association among carriers, negative association among non-carriers).

**Table 3:** Correlation analysis between metabolomics, biochemical and neuropsychological variables within the four study groups stratified according to the ApoE-ε4 genotype and sex (Pearson’s correlation coefficients are shown in brackets). Abbreviations are defined in Table S1

	F_ε4-_	M_ε4-_	F_ε4+_	M_ε4+_
*Biochemical variables*				
Glucose	**FFAs:** C14:0 (0.29), C16:0 (0.33), C16:1 (0.27), C17:0 (0.24), C18:0 (0.19), C18:1 (0.35), C18:2 (0.27), C18:3 (0.37), C20:4 (0.31), C22:4 (0.47), C22:5 (0.28), C22:6 (0.21) **ACs:** C2 (0.28), C3 (0.19), C8 (0.19), C10 (0.19), C12 (0.24), C14 (0.27), C16 (0.22), C18:1 (0.18) **Others:** creatinine (-0.19), lactic acid (0.26), pantothenic acid (0.21)		**FFAs:** C14:0 (0.32), C16:0 (0.41), C17:0 (0.32), C18:0 (0.39), C18:1 (0.30), C18:2 (0.26), C18:3 (0.26), C20:4 (0.35), C22:4 (0.45), C22:5 (0.36), C22:6 (0.26) **ACs:** C2 (0.23) **Others:** creatinine (-0.37), lactic acid (0.26), pyruvic acid (0.28)	**FFAs:** C16:1 (0.43), C18:1 (0.50), C18:3 (0.46)
Creatinine	**ACs:** C2 (0.25), C3 (0.18), C4 (0.28), C11 (0.14), C14 (0.17), C18:2 (0.19) **Others:** creatine (-0.24), pantothenic acid (0.31)	**FFAs:** C16:0 (-0.17), C18:0 (-0.19), C18:1 (-0.18), C20:4 (-0.18), C22:4 (-0.25) **ACs:** C2 (0.25), C3 (0.21), C4 (0.35), C11 (0.18), C14 (0.17)	**ACs:** C8 (0.33), C10 (0.39), C11 (0.41), C12 (0.36) **Others:** pantothenic acid (0.24)	**FFAs:** C18:0 (-0.39) **ACs:** C3 (0.37), C4 (0.56)
Cholesterol	**FFAs:** C22:4 (-0.11) **ACs:** C1 (-0.16), C2 (-0.15) **Others:** pantothenic acid (-0.20)	**FFAs:** C20:4 (0.22), C20:5 (0.19)	**FFAs:** C20:4 (0.42), C22:4 (0.26) **Others:** niacinamide (0.33)	**FFAs:** C17:0 (0.42), C18:2 (0.44)
LDL-C	**FFAs:** C16:1 (-0.14), C22:4 (-0.16) **Others:** pantothenic acid (-0.21)	**FFAs:** C20:4 (0.21) **Others:** niacinamide (0.20), creatine (0.20)	**FFAs:** C20:4 (0.30), **Others:** niacinamide (0.31)	**FFAs:** C17:0 (0.46),
HDL-C	**FFAs:** C18:1 (0.12), C20:4 (0.15), C22:6 (0.13) **ACs:** C8 (0.20), C10 (0.21), C11 (0.22), C12 (0.22)	**FFAs:** C14:0 (0.19) **ACs:** C3 (-0.18), C18:1 (-0.22) **Others:** pantothenic acid (-0.21), niacinamide (-0.24)	**FFAs:** C20:5 (0.34), C22:6 (0.21) **Others:** creatine (0.33)	**Others:** creatinine (-0.42), pyruvic acid (-0.44)
Triglycerides	**FFAs:** C14:0 (0.16), C16:0 (0.15), C22:4 (0.22) **ACs:** C3 (0.18), C14 (0.21), C16 (0.24) **Others:** thiamine (-0.18)	**ACs:** C16 (-0.11) **Others:** niacinamide (0.34), pyruvic acid (0.25), citric acid (-0.23)	**FFAs:** C14:0 (0.38), C16:0 (0.40), C17:0 (0.30), C18:0 (0.34), C18:1 (0.33), C18:2 (0.25), C18:3 (0.28), C20:4 (0.36), C22:4 (0.42), C22:5 (0.30) **Others:** pyruvic acid (0.32)	**FFAs:** C14:0 (0.50), C17:0 (0.41), C18:2 (0.37), C18:3 (0.44), C22:5 (0.38)
*Neuropsychological variables*				
MMSE		**FFAs:** C22:5 (0.19) **ACs:** C8 (0.22), C10 (0.19), C12 (0.23) **Others:** pantothenic acid (-0.20)		**FFAs:** C22:4 (-0.43) **ACs:** C2 (-0.38), C14 (-0.43), C16 (-0.39) **Others:** creatine (0.54), pyruvic acid (-0.44)
BVRT		**Others:** pantothenic acid (-0.22)	**Others:** creatine (0.31)	**Others:** citric acid (0.72)
IST	**FFAs:** C16:0 (-0.14), C16:1 (-0.16), C18:1 (-0.14), C22:4 (-0.14)	**FFAs:** C18:3 (0.17), C20:4 (0.24), C22:4 (0.20), C22:5 (0.19), C22:6 (0.19)	**FFAs:** C22:4 (-0.22)	**FFAs:** C16:0 (0.44), C18:0 (0.54), C18:1 (0.40), C18:2 (0.37), C18:3 (0.40), C20:5 (0.62), C22:6 (0.66) **Others:** citric acid (0.43)
TMTA	**ACs:** C14 (-0.15), C18:1 (-0.15)	**FFAs:** C16:1 (0.19), C18:3 (0.23), C20:4 (0.24), C20:5 (0.28), C22:4 (0.19), C22:5 (0.28), C22:6 (0.33)	**ACs:** C3 (0.31), C4 (0.34), C16 (0.25), C18:1 (0.27)	**FFAs:** C20:4 (0.71), C22:6 (0.43) **Others:** niacinamide (0.46)
TMTB	**FFAs:** C22:4 (-0.13), C22:5 (-0.11) **Others:** citric acid (0.15)	**Others:** niacinamide (-0.22)	**FFAs:** C16:0 (0.30), C18:0 (0.34), C20:4 (0.24) **Others:** citric acid (0.29), lactic acid (0.43)	**Others:** creatinine (-0.58)

Abbreviations: F, female individuals non-carrying the ε4 allele of the apolipoprotein E gene; M_ε4-_, male individuals non-carrying the ε4 allele of the apolipoprotein E gene; F_ε4+_, female individuals carrying the ε4 allele of the apolipoprotein E gene; M_ε4+_, male individuals carrying the ε4 allele of the apolipoprotein E gene; LDL-C, low-density lipoprotein cholesterol; HDL-C, high-density lipoprotein cholesterol; MMSE, Mini-Mental State Examination test; BVRT, Benton Visual Retention Test; IST, Isaac’s Set Test; TMTA, Trail-Making Test part A; TMTB, Trail-Making Test part B; FFAs, free fatty acids; ACs, acyl-carnitines.

## Discussion

**Fig. 1:**
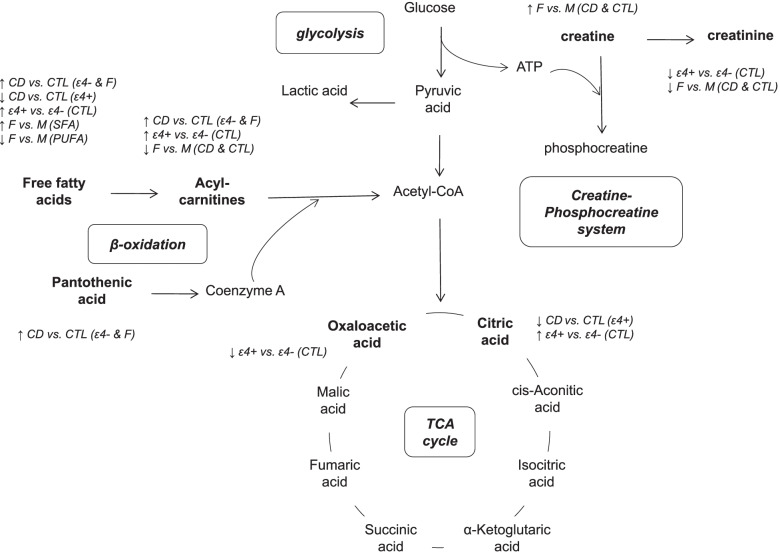
Summary of the ApoE- and sex-modulated metabolic alterations in fatty acid-related pathways during early cognitive decline. Abbreviations: CD, cognitive decline; CTL, control; ε4+, carrier of the ε4 allele of the apolipoprotein E gene; ε4-, non-carrier of the ε4 allele of the apolipoprotein E gene; F, females; M, males; SFA, saturated fatty acids; PUFA; polyunsaturated fatty acids

In this prospective study, metabolomics analysis of serum samples collected at baseline enabled us to explore in detail the association between fatty acid-related metabolites and the subsequent development of CD over a 12-year follow-up, and particularly to investigate how the ApoE-ε4 genotype and sex modify this circulating metabolome linked to CD (Figure 1).

The serum content of FFAs, including saturated and polyunsaturated species, was higher in participants who subsequently developed CD compared to controls while adjusting for the ApoE-ε4 genotype, sex, age, BMI and education level as covariates (Table 2, CD *vs.* CTL). In this respect, it is worth noting that notorious inconsistencies have been reported in previous metabolomics-based studies aimed at investigating the dysregulation of fatty acids in CD and AD [9], which could be attributed to discrepancies in the lipid fraction under study (i.e., FFAs, total fatty acids, fatty acids contained within specific lipid classes). However, when considering the non-esterified fraction, González-Domínguez et al. have consistently found that FFA levels increase in blood from AD patients [23, 24], in line with our results. The accumulation of circulating fatty acids has been linked to an enhanced lipolysis due to the over-activation of phospholipases [1]. In turn, FFAs are thought to participate in the pathogenesis of AD by stimulating the assembly of amyloid plaques and tau filaments [25], as well as by inducing inflammation and insulin resistance [26]. In agreement with recent studies, we furthermore found increased levels of various ACs among CD cases [27–31], suggestive of incomplete fatty acid β-oxidation. A large body of evidence has demonstrated that glucose hypometabolism is a primary hallmark of CD, AD and other neurodegenerative disorders, which starts decades before the onset of clinical symptoms [2]. This bioenergetic malfunction is characterized by reduced glucose metabolism (e.g., glycolysis, pentose phosphate pathway) and impaired mitochondrial function (e.g., oxidative phosphorylation), which consequently provoke a metabolic shift toward the utilization of alternative energy sources (e.g., FFAs, ketone bodies). Accordingly, the accumulation of ACs observed in the present study may possibly occur because the fuel delivered through β-oxidation (i.e., up-regulated fatty acid utilization in an attempt to metabolize the burden of circulating FFAs) exceeds the capacity of energy production by the tricarboxylic acid (TCA) cycle (i.e., incomplete oxidation due to mitochondrial dysfunction), which could result in the retroconversion of acyl-CoA intermediates to ACs and their release into the circulation [32]. This hypothesis is further supported by the increase of pantothenic acid detected in CD subjects, an essential vitamin that serves as the primary precursor of coenzyme A. In this vein, Paglia et al. have reported that high levels of pantothenic acid in AD brains could be indicative of an abnormal homeostasis and transport of acetyl-CoA into the mitochondria and, consequently, impaired brain energy metabolism [33].

In this context, the ApoE-ε4 carrier status and female sex are well-known risk factors for CD and various forms of dementia, which have been demonstrated to be capable of modulating fatty acid metabolism. On the one hand, we found that ε4+ control participants had increased levels of FFAs and ACs compared to their ε4- counterparts, together with significant perturbations in TCA metabolites (i.e., increased citric acid, decreased oxaloacetic acid) (Table 2, CTL_ε4+_ *vs.* CTL_ε4-_). This is in line with other studies conducted in human populations [11, 34], rodent models [35, 36] and astrocyte cultures [37, 38], which all reported that the ε4 allele provokes early and sharpened deficiencies in cerebral glucose metabolism, and consequently promotes the mobilization and oxidation of fatty acids as opposed to using carbohydrates as the energy source. However, the ApoE-ε4 carrier status surprisingly had no significant impact on the fatty acid serum metabolome within CD cases (Table 2, CD_ε4+_ *vs.* CD_ε4-_). Regarding sex, female participants showed increased creatine/creatinine ratio, saturated and monounsaturated FFAs, as well as decreased AC content, when compared to men (Table 2, CTL_F_ *vs.* CTL_M_, CD_F_ *vs.* CD_M_), as previously described by other authors [39–41]. These results could be indicative of higher energy requirements in men due to their generally larger body mass, leading to higher lipid oxidation and energy buffering through the creatine/phosphocreatine system [42]. Conversely, within the control subsample, the concentrations of circulating arachidonic, docosahexaenoic and docosatetraenoic acids were higher in men (Table 2, CTL_F_ *vs.* CTL_M_). This is apparently contradictory with evidence reporting that women show enhanced synthesis of long chain PUFAs, which consequently triggers their accumulation [43]. However, most of the studies previously conducted in this respect were based on the analysis of total lipids and/or specific lipid fractions (e.g., phospholipids), which could account for at least some of the discrepancies reported here. Therefore, considering the great impact of ApoE and sex on the circulating metabolome, stratified analyses were performed with the aim of obtaining deeper insights into the interactions between CD and these risk factors.

The stratification of the study population according to the ApoE-ε4 genotype revealed that the CD-related increase described above in the serum content of FFAs, ACs and pantothenic acid was specific for the non-carrier sub-sample (Table 2, CD_ε4-_ *vs.* CTL_ε4-_). In contrast, subjects with the ε4+ genotype surprisingly showed reduced circulating concentrations of various FFAs and citric acid, whereas the other metabolite classes remained unchanged (Table 2, CD_ε4+_ *vs.* CTL_ε4+_). Similarly, sex also elicited a differential impact on these metabolomics alterations, since the accumulation of FFAs, ACs and pantothenic acid among CD cases was only detected within women (Table 2, CD_F_ *vs.* CTL_F_), although the same trend was observed within males for ACs and pantothenic acid without reaching statistical significance (Table 2, CD_M_ *vs.* CTL_M_). Altogether, these results suggest that the modulation of fatty acid metabolism and related pathways in the onset of CD depends on complex inter-relationships between the ApoE-ε4 genotype and sex. In this vein, previous studies have already demonstrated that CD patients carrying the ε4 allele are more resistant to dietary (e.g., omega-3 enriched diets) and drug-based (e.g., peroxisome proliferator-activated receptor gamma agonists) interventions designed to mitigate the negative effects of ApoE-ε4 on fatty acid metabolism [44]. Recently, Lim et al. described that the association between plasma lipids and AD is stronger within women and subjects without the ɛ4 allele [11]. So far, the mechanisms behind the interactions between ApoE-ε4 and sex on the homeostasis of fatty acids remain to be elucidated. Accordingly, to better explore the effect of ApoE and sex on CD and associated alterations in circulating fatty acids and related metabolites, Pearson’s correlations were computed between metabolomics, biochemical and neuropsychological variables using stratified analyses (Table 3). Notably, only plasma creatinine and various ACs were consistently correlated in the four study sub-groups, in line with previous studies reporting that these metabolites participate in metabolic pathways that are closely interrelated (i.e., creatine/phosphocreatine system and β-oxidation, respectively), and therefore could serve as indicators of mitochondrial dysfunction in CD and AD [28, 45]. However, the rest of the variables studied here showed heterogeneous associations depending on the ApoE genotype and/or sex. Interestingly, sex-specific associations were found between fatty acid and energy-related metabolites, glucose and creatinine, indicating that energy metabolism could be differentially impaired in the onset of CD depending on sex. On one hand, plasma glucose was positively correlated with FFAs, ACs and glycolytic end-products (i.e., lactic acid, pyruvic acid), but negatively with creatinine levels, among women. In contrast, creatinine and circulating FFAs showed an inverse association among men. In line with findings by Arnold et al., our results suggest that glucose hypometabolism could be closely related to impaired energy production via mitochondrial β-oxidation and glycolysis in women, whereas men appear to compensate the energy dysfunctions by switching to the provision of fatty acids as alternative fuel sources [10]. Our study thus reinforces the idea that females experience greater impairments of mitochondrial energy production than males, with fatty acid oxidation being at least partially sustained in men during early CD. Besides these associations among energy-related metabolites, the levels of various FFAs and ACs were also significantly correlated to plasma cholesterol and triglycerides (Table 3). Overall, FFAs were positively associated with total cholesterol, LDL-C, HDL-C and triglycerides, which could be considered a direct indicator of the characteristic dyslipidemia observed in CD and dementia [46]. However, the opposite direction of association was curiously found between FFAs, total cholesterol and LDL-C, indicating an intertwined modulation of cholesterol metabolism by the ApoE-ε4 genotype and female sex [3]. Finally, Pearson’s correlation analysis showed differential associations between serum metabolites and neuropsychological variables depending on the ApoE carrier status and sex. Circulating FFAs were correlated with the MMSE and TMTA scores only within men, whereas the association with TMTB was specific for females. In contrast, the IST score was correlated with FFAs in a sex-dependent manner (positive correlation among males, negative correlation among females). On the other hand, ACs showed sex-specific and ApoE-dependent associations with MMSE and TMTA scores, as shown in Table 3. Altogether, these results reinforce that CD-triggered impairments in the different cognitive domains are modulated in a sex and ApoE dependent manner.

## Conclusions

In summary, this study demonstrates that the early onset of CD may possibly be preceded by profound alterations in fatty acid metabolism and related metabolic pathways, which are in turn modulated by ApoE-ε4 and sex. In particular, we found that various circulating serum metabolites, including free fatty acids, acyl-carnitines and other energy-related metabolites, were associated with CD in an ApoE and/or sex dependent manner. These heterogeneous results between subgroups stratified according to the ApoE-ε4 carrier status and sex highlight the great impact of these common risk factors for CD on the human metabolome and, consequently, the crucial importance of adequately addressing the inter-individual variability in metabolomics research. In fact, this heterogeneity could explain, at least in part, the inconsistencies repeatedly reported in previous metabolomics studies on CD, AD and related disorders [9]. Accordingly, the present study emphasizes the need for further investigating the within-group differences triggered by inter-individual variability factors (i.e., metabotyping), which in turn would facilitate the development of more effective preventive, diagnostic and treatment approaches in the context of precision medicine.

## Limitations

A major strength of this study is the well characterized long-term prospective cohort of older subjects over a 12-year follow-up. The application of a targeted metabolomics approach to serum samples collected at baseline, prior to the appearance of dementia symptoms, enabled us to elucidate the intertwined modulation of the fatty acid-related metabolome by ApoE-ε4 and sex at very early stages of CD, and thereby complement previous studies conducted in AD populations [10–12]. However, it should be noted that stratification of the study population may inherently limit the statistical power of the stratified analyses. This is especially relevant as CD cases and controls were poorly balanced for the ApoE-ε4 status and sex, and each of the subgroups under study had different sample sizes. Furthermore, it is also noteworthy that the study design was limited to individual from the Bordeaux center of the 3C study, which might bias the results (e.g., dietary habits). Future studies in larger and independent populations are needed to further validate our findings, better characterize the involvement of fatty acid and energy-related metabolism in CD and dementia, and associate these metabolic factors with additional genetic, neuroimaging and biochemical data (e.g., β amyloid, tau). Longitudinal studies, with metabolomics and neuropsychological data collected along the follow-up period, would be of great interest to investigate the progression of these disorders.

## Supplementary Information

**Additional file 1: Table S1.** List of metabolites analyzed in serum samples. **Table S2.** Details about the ApoE stratification of the study population. **Table S3.** Average neuropsychological test scores at each visit along the follow-up (i.e., V = 0, 2, 4, 7, 10 and 12 years) in cases and matched controls individuals. Values are expressed as mean (SD). **Table S4.** Concentrations within each study group of the fatty acid-related metabolites that were identified by lineal modeling to be associated with cognitive decline, ApoE-ε4 genotype and sex. The results are expressed as mean (SD) in μg L^-1^. **Table S5.** Concentrations within ApoE-stratified subgroups of the fatty acid-related metabolites that were identified by lineal modeling to be associated with cognitive decline, ApoE-ε4 genotype and sex. The results are expressed as mean (SD) in μg L^-1^. **Figure S1.** Pearson’s correlation analysis between metabolomics, biochemical and neuropsychological variables within the four study groups stratified according to the ApoE-ε4 genotype and sex: female non-carriers (A), male non-carriers (B), female carriers (C), and male carriers (D).

## Data Availability

The datasets used and/or analyzed during the current study are available from the corresponding author on reasonable request.

## Abbreviations

AC: Acyl-carnitine
AD: Alzheimer’s disease
ApoE: Apolipoprotein E
BVRT: Benton Visual Retention Test
CD: Cognitive decline
CDε4+: Cognitive decline subjects carrying the ApoE-ε4 allele
CDε4-: Cognitive decline subjects non-carrying the ApoE-ε4 allele
CDF: Cognitive decline female subjects
CDM: Cognitive decline male subjects
CTL: Control
CTLε4+: Control subjects carrying the ApoE-ε4 allele
CTLε4-: Control subjects non-carrying the ApoE-ε4 allele
CTLF: Control female subjects
CTLM: Control male subjects;
Fε4+: Female subjects carrying the ApoE-ε4 allele
Fε4-: Female subjects non-carrying the ApoE-ε4 allele
FFA: Free fatty acid
IST: Isaac’s Set Test
Mε4+: Male subjects carrying the ApoE-ε4 allele
Mε4-: Male subjects non-carrying the ApoE-ε4 allele
MCI: Mild cognitive impairment
MMSE: Mini-Mental State Examination test
PUFA: Polyunsaturated fatty acid
TCA: Tricarboxylic acid
TMTA: Trail-Making Test part A
TMTB: Trail-Making Test part B
