# Supplementary material for: Apolipoprotein E and sex modulate fatty acid metabolism in a prospective observational study of cognitive decline
Source: Alzheimers Res Ther. 2022 Jan 3;14:1. doi: 10.1186/s13195-021-00948-8 (PMC8725342; doi:10.1186/s13195-021-00948-8)
Supplement: Supplementary file 1 — Additional file 1: Table S1. List of metabolites analyzed in serum samples. Table S2. Details about the ApoE stratification of the study population. Table S3. Average neuropsychological test scores at each visit along the follow-up (i.e., V = 0, 2, 4, 7, 10 and 12 years) in cases and matched controls individuals. Values are expressed as mean (SD). Table S4. Concentrations within each study group of the fatty acid-related metabolites that were identified by lineal modeling to be associated with cognitive decline, ApoE-ε4 genotype and sex. The results are expressed as mean (SD) in μg L-1. Table S5. Concentrations within ApoE-stratified subgroups of the fatty acid-related metabolites that were identified by lineal modeling to be associated with cognitive decline, ApoE-ε4 genotype and sex. The results are expressed as mean (SD) in μg L-1. Figure S1. Pearson’s correlation analysis between metabolomics, biochemical and neuropsychological variables within the four study groups stratified according to the ApoE-ε4 genotype and sex: female non-carriers (A), male non-carriers (B), female carriers (C), and male carriers (D). [file 13195_2021_948_MOESM1_ESM.docx]

**Apolipoprotein E and sex modulate fatty acid metabolism in a prospective observational study of cognitive decline**

Raúl González-Domínguez^1,2,*^, Pol Castellano-Escuder^1,2,3^, Sophie Lefèvre-Arbogast^4^, Dorrain Y. Low^5^, Andrea Du Preez^6^, Silvie R. Ruigrok^7^, Hyunah Lee^6^, Catherine Helmer^4^, Mercè Pallàs^8^, Mireia Urpi-Sarda^1,2^, Alex Sánchez-Pla^2,3^, Aniko Korosi^7^, Paul J. Lucassen^7^, Ludwig Aigner^9^, Claudine Manach^5^, Sandrine Thuret^6^, Cécilia Samieri^4^, Cristina Andres-Lacueva^1,2,*^

^1^Biomarkers and Nutrimetabolomics Laboratory, Food Innovation Network (XIA), Nutrition and Food Safety Research Institute (INSA), Faculty of Pharmacy and Food Sciences, University of Barcelona, 08028 Barcelona, Spain. ^2^CIBER Fragilidad y Envejecimiento Saludable (CIBERfes), Instituto de Salud Carlos III, 28029 Madrid, Spain. ^3^Department of Genetics, Microbiology and Statistics, University of Barcelona, 08028 Barcelona, Spain. ^4^University of Bordeaux, Inserm, Bordeaux Population Health Research Center, UMR 1219, F-33000 Bordeaux, France. ^5^Université Clermont Auvergne, INRAE, UNH, F-63000 Clermont Ferrand, France. ^6^Department of Basic and Clinical Neuroscience, Maurice Wohl Clinical Neuroscience Institute, Institute of Psychiatry, Psychology and Neuroscience, King’s College London, London SE5 9NU, UK. ^7^Brain Plasticity Group, Swammerdam Institute for Life Sciences, Center for Neuroscience, University of Amsterdam, 1098 XH Amsterdam, The Netherlands. ^8^Pharmacology Section, Department of Pharmacology, Toxicology and Medicinal Chemistry, Faculty of Pharmacy and Food Sciences, and Institut de Neurociències, University of Barcelona, 08028 Barcelona, Spain. ^9^Institute of Molecular Regenerative Medicine, Spinal Cord Injury and Tissue Regeneration Center Salzburg, Paracelsus Medical University, Salzburg 5020, Austria.

*Corresponding authors: Dr. Raúl González-Domínguez, Tel. +34-934024513, E-mail: raul.gonzalez@ub.edu; Prof. Cristina Andres-Lacueva, Tel. +34-934034840, E-mail: candres@ub.edu

**SUPPLEMENTARY MATERIAL**

**Table S1.** List of metabolites analyzed in serum samples.

**Table S2.** Details about the ApoE stratification of the study population.

**Table S3.** Average neuropsychological test scores at each visit along the follow-up (i.e., V = 0, 2, 4, 7, 10 and 12 years) in cases and matched controls individuals. Values are expressed as mean (SD).

**Table S4.** Concentrations within each study group of the fatty acid-related metabolites that were identified by lineal modeling to be associated with cognitive decline, ApoE-ε4 genotype and sex. The results are expressed as mean (SD) in µg L^-1^.

**Table S5.** Concentrations within ApoE-stratified subgroups of the fatty acid-related metabolites that were identified by lineal modeling to be associated with cognitive decline, ApoE-ε4 genotype and sex. The results are expressed as mean (SD) in µg L^-1^.

**Figure S1.** Pearson’s correlation analysis between metabolomics, biochemical and neuropsychological variables within the four study groups stratified according to the ApoE-ε4 genotype and sex: female non-carriers (A), male non-carriers (B), female carriers (C), and male carriers (D).

**Table S1.** List of metabolites analyzed in serum samples.

| **Free fatty acids** | **Acyl-carnitines** | **Other energy-related metabolites** | **B-group vitamins** |
| --- | --- | --- | --- |
| Octanoic acid (C8:0) | L-carnitine (C1) | Glucose | Thiamine |
| Decanoic acid (C10:0) | Acetyl-L-carnitine (C2) | Pyruvic acid | Riboflavin |
| Lauric acid (C12:0) | Propionyl-L-carnitine (C3) | Lactic acid | Niacin |
| Myristic acid (C14:0) | Butyryl-L-carnitine (C4) | Citric acid | Niacinamide |
| Pentadecanoic acid (C15:0) | Isobutyryl-L-carnitine (iC4) | cis-Aconitic acid | Pantothenic acid |
| Palmitic acid (C16:0) | Valeryl-L-carnitine (C5) | α-Ketoglutaric acid | Pyridoxal |
| Palmitoleic acid (C16:1) | Hexanoyl-L-carnitine (C6) | Succinic acid | Pyridoxal 5-phosphate |
| Margaric acid (17:0) | Octanoyl-L-carnitine (C8) | Fumaric acid | 4-Pyridoxic acid |
| Stearic acid (C18:0) | Octenoyl-L-carnitine (C8:1) | Malic acid | Biotin |
| Oleic acid (C18:1) | Decanoyl-L-carnitine (C10) | Oxaloacetic acid |  |
| Linoleic acid (C18:2) | Undecanoyl-L-carnitine (C11) | Creatine |  |
| Linolenic acid (C18:3) | Lauroyl-L-carnitine (C12) | Creatinine |  |
| Arachidonic acid (C20:4) | Myristoyl-L-carnitine (C14) |  |  |
| Eicosapentaenoic acid (C20:5) | Palmitoyl-L-carnitine (C16) |  |  |
| Docosatetraenoic acid (C22:4) | Stearoyl-L-carnitine (C18) |  |  |
| Docosapentaenoic acid (C22:5) | Oleoyl-L-carnitine (C18:1) |  |  |
| Docosahexaenoic acid (C22:6) | Linoleoyl-L-carnitine (C18:2) |  |  |

**Table S2.** Details about the ApoE stratification of the study population.

|  | **Controls** | **Cases** | **All** |
| --- | --- | --- | --- |
| ε2/ε2 | 0 | 1 | 1 |
| ε2/ε3 | 25 | 29 | 54 |
| ε3/ε3 | 121 | 120 | 241 |
| ε2/ε4 | 0 | 2 | 2 |
| ε3/ε4 | 20 | 41 | 61 |
| ε4/ε4 | 0 | 9 | 9 |

**Table S3.** Average neuropsychological test scores at each visit along the follow-up (i.e., V = 0, 2, 4, 7, 10 and 12 years) in cases and matched controls individuals. Values are expressed as mean (SD).

|  | **V0 (Baseline)** | **V2** | **V4** | **V7** | **V10** | **V12** |
| --- | --- | --- | --- | --- | --- | --- |
| **MMSE, points (range 0-30)** | | | | | | |
| Cases | 26.9 (2.2) | 27.0 (2.2) | 26.6 (2.5) | 24.2 (4.6) | 21.4 (4.7) | 16.3 (7.0) |
| Controls | 28.0 (1.6) | 28.0 (1.7) | 28.1 (1.5) | 28.4 (1.4) | 27.9 (1.5) | 28.0 (2.0) |
| **BVRT, points (range 0-15)** | | | | | | |
| Cases | 10.8 (2.1) | 10.7 (2.2) | 10.5 (2.3) | 9.0 (2.4) | 7.9 (2.4) | 7.6 (2.4) |
| Controls | 11.7 (1.9) | 12.1 (2.0) | 11.9 (1.7) | 12.3 (1.6) | 11.8 (1.8) | 12.0 (1.4) |
| **IST, points (range 0-40)** | | | | | | |
| Cases | 27.7 (6.0) | 28.8 (6.0) | 26.5 (6.7) | 22.1 (5.9) | 18.6 (5.7) | 13.1 (7.0) |
| Controls | 31.0 (6.1) | 33.0 (5.7) | 33.9 (6.0) | 31.0 (5.3) | 28.9 (5.1) | 29.4 (5.0) |
| **TMTA, number of correct displacements per minute** | | | | | | |
| Cases | 24.0 (7.9) | - | 21.7 (8.2) | 18.2 (7.2) | 13.8 (6.0) | 7.6 (5.4) |
| Controls | 29.4 (9.1) | - | 30.8 (10.4) | 29.6 (9.5) | 26.6 (8.7) | 27.0 (10.2) |
| **TMTB, number of correct displacements per minute** | | | | | | |
| Cases | 10.0 (5.7) | - | 7.8 (5.3) | 5.7 (3.8) | 3.4 (2.5) | 1.6 (0.9) |
| Controls | 14.5 (6.5) | - | 13.5 (6.5) | 14.0 (6.2) | 10.9 (5.2) | 10.9 (5.5) |

Abbreviations: MMSE, Mini-Mental State Examination test; BVRT, Benton Visual Retention Test; IST, Isaac’s Set Test; TMTA, Trail-Making Test part A; TMTB, Trail-Making Test part B.

**Table S4.** Concentrations within each study group of the fatty acid-related metabolites that were identified by lineal modeling to be associated with cognitive decline, ApoE-ε4 genotype and sex. The results are expressed as mean (SD) in µg L^-1^.

|  | Whole study population | | ApoE-ε4 stratification | | | | Sex stratification | | | |
| --- | --- | --- | --- | --- | --- | --- | --- | --- | --- | --- |
|  | CD | CTL | CD_ε4+_ | CD_ε4-_ | CTL_ε4+_ | CTL_ε4-_ | CD_F_ | CD_M_ | CTL_F_ | CTL_M_ |
| *Free fatty acids* | | | | | | | | | | |
| Myristic acid | 19367.4 (8462.0) | 17794.1 (7462.8) | 12282.9 (12383.9) | 17030.0 (10966.1) | 16596.7 (12449.6) | 13990.2 (10152.6) | 18818.6 (10835.4) | 14420.5 (11767.8) | 15152.6 (10751.1) | 12600.9 (9709.6) |
| Palmitic acid | 35697.8 (14056.5) | 33563.1 (12040.9) | 33823.6 (11863.1) | 36347.6 (14721.4) | 43573.9 (13634.0) | 32191.7 (11176.1) | 36412.3 (15430.5) | 34320.6 (10904.9) | 33061.5 (12099.5) | 34548.2 (11971.8) |
| Palmitoleic acid | 19637.4 (8462.0) | 17794.1 (7462.8) | 18983.9 (7551.2) | 19500.3 (8775.5) | 22893.4 (8565.6) | 17095.6 (7048.7) | 20594.3 (9085.9) | 17002.5 (6541.0) | 18430.0 (7824.5) | 16454.2 (6584.1) |
| Margaric acid | 5728.0 (2291.1) | 5346.4 (2204.4) | 5832.4 (2426.6) | 5691.8 (2249.5) | 6312.5 (3064.4) | 5214.1 (2037.8) | 5805.7 (2388.7) | 5578.2 (2098.7) | 5371.0 (2363.8) | 5298.1 (1871.1) |
| Stearic acid | 29454.4 (20168.5) | 28562.5 (18006.6) | 25593.9  (11571.8) | 30792.7 (22268.5) | 40442.5 (21309.7) | 26935.1 (16950.6) | 30180.2 (22746.0) | 28055.3 (13964.8) | 28501.7 (20006.0) | 28681.9 (13397.4) |
| Oleic acid | 70226.7 (27168.6) | 64201.8 (25104.8) | 65204.4 (23706.9) | 71967.7 (28133.9) | 83759.2 (25465.6) | 61522.7 (23921.2) | 72735.8 (29999.1) | 65390.1 (19970.6) | 64138.3 (25050.8) | 64326.4 (25437.2) |
| Linoleic acid | 51808.2 (16036.2) | 47396.5 (14845.7) | 51364.9 (16561.9) | 51961.8 (15903.7) | 55672.0 (20641.2) | 46262.9 (13573.0) | 52111.2 (16885.1) | 51224.1 (14358.6) | 46783.2 (15708.2) | 48601.2 (13036.6) |
| Linolenic acid | 6934.0 (2304.7) | 6543.6 (2133.4) | 6728.8 (2172.7) | 7005.1 (2351.5) | 7836.1 (2652.1) | 6366.6 (1998.9) | 7208.2 (2515.9) | 6405.4 (1727.0) | 6502.5 (2203.6) | 6624.5 (2005.3) |
| Arachidonic acid | 7349.0 (2227.2) | 7135.8 (1997.0) | 7289.6 (2115.3) | 7369.7 (2271.2) | 8567.6 (2041.1) | 6939.7 (1915.9) | 7360.4 (2365.1) | 7327.1 (1950.4) | 6914.3 (1942.4) | 7570.8 (2048.4) |
| Eicosapentaenoic acid | 2986.1 (746.3) | 2986.6 (674.3) | 2907.2 (450.5) | 3013.5 (824.0) | 3316.0 (1187.8) | 2941.5 (561.7) | 2995.9 (796.7) | 2967.2 (643.1) | 2908.7 (525.6) | 3139.7 (883.4) |
| Docosatetraenoic acid | 3733.8 (903.1) | 3489.9 (741.7) | 3753.1 (1006.1) | 3727.1 (868.0) | 4056.9 (900.0) | 3412.2 (685.2) | 3763.6 (970.4) | 3676.4 (760.0) | 3404.9 (710.3) | 3656.8 (779.6) |
| Docosapentaenoic acid | 14092.7 (6589.9) | 12377.2 (4820.3) | 14114.5 (5809.4) | 14085.2 (6857.9) | 15413.6 (6069.4) | 11961.3 (4490.9) | 14368.1 (7349.4) | 13562.0 (8404.3) | 11997.9 (4721.4) | 13122.3 (4967.5) |
| Docosahexaenoic acid | 6566.3 (2313.5) | 6320.6 (2100.0) | 6227.1 (1711.7) | 6683.8 (2482.6) | 7590.9 (2881.5) | 6146.6 (1917.6) | 6661.6 (2527.6) | 6382.5 (1835.0) | 6060.4 (1900.2) | 6831.8 (2381.7) |
| *Acyl-carnitines* | | | | | | | | | | |
| Acetyl-L-carnitine | 30.6 (16.2) | 24.9 (9.0) | 30.0 (12.6) | 30.8 (17.3) | 30.2 (9.4) | 24.1 (8.7) | 31.0 (18.1) | 29.9 (11.9) | 25.0 (9.7) | 24.7 (7.4) |
| Octanoyl-L-carnitine | 6.2 (7.1) | 4.5 (2.8) | 5.2 (3.4) | 6.6 (8.0) | 5.2 (2.8) | 4.4 (2.8) | 6.6 (8.5) | 5.5 (3.1) | 4.4 (2.9) | 4.7 (2.7) |
| Decanoyl-L-carnitine | 15.8 (12.3) | 12.2 (7.0) | 13.4 (8.2) | 16.6 (13.3) | 13.8 (6.7) | 12.0 (7.0) | 16.4 (14.1) | 14.6 (7.5) | 12.0 (7.3) | 12.7 (6.5) |
| Undecanoyl-L-carnitine | 1.1 (0.3) | 1.0 (0.2) | 1.1 (0.2) | 1.1 (0.3) | 1.1 (0.1) | 1.0 (0.2) | 1.1 (0.3) | 1.1 (0.2) | 1.0 (0.2) | 1.0 (0.2) |
| Lauroyl-L-carnitine | 16.7 (9.7) | 13.3 (6.4) | 15.6 (8.5) | 17.0 (10.1) | 15.9 (5.6) | 12.9 (6.4) | 17.0 (11.0) | 16.1 (6.6) | 12.8 (6.3) | 14.3 (6.3) |
| Myristoyl-L-carnitine | 12.7 (4.6) | 10.9 (3.0) | 12.8 (4.4) | 12.7 (4.7) | 12.4 (1.8) | 10.7 (3.1) | 12.8 (5.0) | 12.6 (3.8) | 10.8 (3.2) | 11.1 (2.7) |
| Palmitoyl-L-carnitine | 29.0 (8.6) | 25.9 (6.7) | 30.3 (9.8) | 28.6 (8.2) | 28.3 (5.2) | 25.6 (6.9) | 28.3 (8.7) | 30.5 (8.4) | 25.1 (6.8) | 27.7 (6.3) |
| Oleoyl-L-carnitine | 113.3 (144.1) | 98.6 (35.0) | 112.8 (47.5) | 113.4 (43.1) | 111.5 (28.5) | 96.9 (35.5) | 111.9 (46.3) | 116.0 (39.7) | 96.0 (35.6) | 103.8 (33.3) |
| Linoleoyl-L-carnitine | 89.4 (39.8) | 76.6 (34.6) | 92.4 (41.1) | 88.4 (39.5) | 39.5 (79.9) | 76.2 (36.1) | 83.0 (40.5) | 101.8 (35.6) | 69.0 (28.2) | 91.6 (40.7) |
| *Other metabolites* | | | | | | | | | | |
| Citric acid | 5702.1 (5365.9) | 5561.9 (5266.9) | 4340.8 (3653.2) | 6149.4 (5760.7) | 8141.9 (5677.1) | 5196.1 (5124.1) | 5925.6 (5686.2) | 5265.7 (4690.3) | 5809.3 (5537.6) | 5081.5 (4711.1) |
| Oxaloacetic acid | 17994.2 (21754.6) | 16616.0 (20644.2) | 20251.6 (21617.0) | 17211.6 (21819.5) | 7839.3 (13865.7) | 17818.3 (21158.8) | 17300.3 (21787.1) | 19331.5 (21788.1) | 16730.2 (20787.1) | 16391.5 (20545.7) |
| Creatine | 10667.0 (5328.0) | 10712.1 (4924.8) | 9543.0 (4482.1) | 11056.6 (5552.0) | 12598.2 (5479.8) | 10453.7 (4806.9) | 12116.5 (5209.4) | 7873.0 (7382.0) | 12314.6 (4436.8) | 7564.3 (4304.0) |
| Creatinine | 873.5 (193.3) | 850.3 (180.9) | 878.1 (199.9) | 871.9 (191.6) | 768.8 (167.3) | 861.4 (180.4) | 808.8 (152.2) | 998.2 (203.6) | 807.0 (168.7) | 935.3 (175.2) |
| Pantothenic acid | 62.6 (33.9) | 52.3 (24.8) | 68.0 (38.9) | 60.7 (31.9) | 57.7 (28.4) | 51.6 (24.3) | 64.4 (37.0) | 59.0 (26.9) | 52.7 (25.9) | 51.5 (22.7) |

Abbreviations: CD, cognitive decline; CTL, control; CD_ε4+_, cognitive decline individuals carrying the ε4 allele of the apolipoprotein E gene; CD_ε4-_, cognitive decline individuals non-carrying the ε4 allele of the apolipoprotein E gene; CTL_ε4+_, control individuals carrying the ε4 allele of the apolipoprotein E gene; CTL_ε4-_, control individuals non-carrying the ε4 allele of the apolipoprotein E gene; CD_F_, cognitive decline female individuals; CD_M_, cognitive decline male individuals; CTL_F_, control female individuals; CTL_M_, control male individuals.

**Table S5.** Concentrations within ApoE-stratified subgroups of the fatty acid-related metabolites that were identified by lineal modeling to be associated with cognitive decline, ApoE-ε4 genotype and sex. The results are expressed as mean (SD) in µg L^-1^.

|  | cases | | | | | | controls | | |
| --- | --- | --- | --- | --- | --- | --- | --- | --- | --- |
|  | ε2/ε2 (N = 1) | ε2/ε3 (N = 29) | ε2/ε4 (N = 2) | ε3/ε3 (N = 120) | ε3/ε4 (N = 41) | ε4/ε4 (N = 9) | ε2/ε3 (N = 25) | ε3/ε3 (N = 121) | ε3/ε4 (N = 20) |
| *Free fatty acids* | | | | | | | | | |
| Myristic acid | 10558.1 | 17458.3 (11648.9) | 12896.9 (12383.9) | 16981.6 (10875.5) | 16964.5 (11927.6) | 20383.7 (8456.3) | 16363.3 (11891.9) | 13495.0 (9737.1) | 16596.7 (12449.6) |
| Palmitic acid | 34886.6 | 37189.9 (14310.4) | 33776.3 (18672.3) | 36156.2 (14931.2) | 33632.8 (11445.5) | 34703.3 (14054.8) | 31442.7 (10875.4) | 32346.5 (11275.3) | 43573.9 (13634.0) |
| Palmitoleic acid | 12737.6 | 20251.5 (8371.3) | 16693.1 (9449.5) | 19375.1 (8910.4) | 18211.2 (6937.3) | 22346.5 (9788.1) | 18478.1 (9576.6) | 16809.9 (6419.6) | 22893.4 (8565.6) |
| Margaric acid | 9307.2 | 5658.5 (2730.3) | 7637.2 (6673.9) | 5669.7 (2114.5) | 5665.9 (2296.3) | 6189.8 (2122.9) | 5649.7 (2277.6) | 5124.1 (1983.1) | 6312.5 (3064.4) |
| Stearic acid | 36591.0 | 29184.9 (14726.4) | 19612.9  (2341.5) | 31132.9 (23850.5) | 25125.3 (10555.9) | 29057.8 (16500.7) | 24851.2 (8430.3) | 27365.7 (18217.5) | 40442.5 (21309.7) |
| Oleic acid | 53223.1 | 74594.8 (28799.4) | 66250.0 (35098.3) | 71489.0 (28125.4) | 64890.7 (23307.0) | 66401.2 (26653.2) | 64069.7 (30199.1) | 60996.5 (22525.7) | 83759.2 (25465.6) |
| Linoleic acid | 63188.6 | 53090.0 (14470.2) | 48645.7 (23289.1) | 51595.7 (15600.5) | 52122.3 (17653.9) | 48518.9 (10423.1) | 47775.4 (15664.1) | 45950.4 (13151.4) | 55672.0 (20641.2) |
| Linolenic acid | 6115.4 | 7024.5 (2175.8) | 6896.1 (1051.1) | 7007.8 (2409.0) | 6842.8 (2322.5) | 6172.7 (1601.9) | 6427.0 (2168.3) | 6354.1 (1971.5) | 7836.1 (2652.1) |
| Arachidonic acid | 4356.6 | 7282.8 (1929.3) | 6848.6 (628.5) | 7415.7 (2345.8) | 7538.9 (2180.8) | 6251.9 (1765.5) | 6903.2 (2098.9) | 6947.2 (1885.3) | 8567.6 (2041.1) |
| Eicosapentaenoic acid | 2480.5 | 2892.4 (388.8) | 2920.3 (521.7) | 3047.2 (898.6) | 2909.3 (461.2) | 2894.8 (442.5) | 2800.7  (385.6) | 2970.6 (588.6) | 3316.0 (1187.8) |
| Docosatetraenoic acid | 3099.1 | 3921.1 (1107.6) | 3737.3 (1105.6) | 3685.5 (800.4) | 3752.6 (970.6) | 3758.8 (1261.0) | 3388.7 (725.0) | 3417.0 (679.7) | 4056.9 (900.0) |
| Docosapentaenoic acid | 15208.2 | 13878.6 (7321.1) | 18440.1 (13507.4) | 14125.7 (6801.0) | 13970.5 (5529.9) | 13809.4 (5878.7) | 12035.7 (4859.9) | 11945.9 (4432.2) | 15413.6 (6069.4) |
| Docosahexaenoic acid | 5846.3 | 6411.8 (1576.5) | 7125.6 (889.2) | 6756.6 (2665.2) | 6192.1 (1713.8) | 6186.8 (1918.9) | 5875.4 (1712.5) | 6202.7 (1959.2) | 7590.9 (2881.5) |
| *Acyl-carnitines* | | | | | | | | | |
| Acetyl-L-carnitine | 10.3 | 30.1 (13.2) | 31.5 (16.4) | 31.2 (18.2) | 29.6 (12.5) | 31.7 (13.8) | 28.9 (10.8) | 23.2 (8.0) | 30.2 (9.4) |
| Octanoyl-L-carnitine | 87.5 | 5.5 (3.3) | 5.2 (4.8) | 6.1 (4.7) | 5.3 (3.4) | 4.7 (3.5) | 5.3 (3.3) | 4.2 (2.7) | 5.2 (2.8) |
| Decanoyl-L-carnitine | 119.2 | 14.7 (8.2) | 13.9 (11.9) | 16.2 (10.8) | 13.3 (8.0) | 13.8 (9.8) | 13.9 (8.1) | 11.6 (6.8) | 13.8 (6.7) |
| Undecanoyl-L-carnitine | 2.9 | 1.1 (0.2) | 1.3 (0.6) | 1.1 (0.3) | 1.1 (0.2) | 1.1 (0.2) | 1.1 (0.0) | 1.0 (0.2) | 1.1 (0.1) |
| Lauroyl-L-carnitine | 56.2 | 15.7 (8.3) | 19.1 (15.2) | 17.0 (9.9) | 15.0 (8.0) | 17.9 (10.1) | 14.4 (7.0) | 12.6 (6.3) | 15.9 (5.6) |
| Myristoyl-L-carnitine | 22.9 | 12.1 (3.8) | 15.8 (10.2) | 12.7 (4.8) | 12.3 (3.9) | 14.3 (5.2) | 11.3 (3.7) | 10.6 (3.0) | 12.4 (1.8) |
| Palmitoyl-L-carnitine | 25.7 | 28.1 (7.3) | 30.8 (10.0) | 28.7 (8.4) | 30.1 (9.8) | 31.2 (10.8) | 26.5 (8.4) | 25.4 (6.5) | 28.3 (5.2) |
| Oleoyl-L-carnitine | 92.0 | 111.7 (41.1) | 99.5 (2.4) | 114.0 (43.8) | 113.6 (50.8) | 112.2 (37.5) | 100.3 (45.9) | 96.1 (33.1) | 111.5 (28.5) |
| Linoleoyl-L-carnitine | 76.5 | 90.5 (40.0) | 70.6 (28.6) | 88.0 (39.7) | 96.3 (42.5) | 79.3 (34.9) | 76.9 (43.1) | 76.0 (34.6) | 79.9 (20.7) |
| *Other metabolites* | | | | | | | | | |
| Citric acid | 81.1 | 7182.9 (6053.1) | 1173.6 (1553.2) | 5965.3 (5691.8) | 4381.6 (3743.1) | 4548.1 (3499.7) | 5950.3 (5505.1) | 5048.0 (5058.8) | 8141.9 (5677.1) |
| Oxaloacetic acid | 42025.6 | 15052.4 (18973.5) | 51826.1 (13693.6) | 17526.6 (22472.1) | 17974.3 (20331.4) | 23609.5 (24499.6) | 21122.7 (23191.8) | 17135.5 (20752.1) | 7839.3 (13865.7) |
| Creatine | 15074.9 | 9510.5 (4653.7) | 8501.3 (7569.6) | 11396.8 (5715.4) | 10028.1 (4320.6) | 7564.3 (4657.0) | 11355.7 (4738.1) | 10267.4 (4819.3) | 12598.2 (5479.8) |
| Creatinine | 752.9 | 890.8 (226.9) | 813.9 (379.4) | 868.3 (183.3) | 887.9 (212.0) | 847.6 (97.6) | 855.8 (155.9) | 862.6 (185.6) | 768.8 (167.3) |
| Pantothenic acid | 37.7 | 63.4 (28.4) | 48.4 (12.4) | 60.2 (32.9) | 70.1 (43.0) | 63.1 (14.5) | 60.3 (33.6) | 49.7 (21.6) | 57.7 (28.4) |


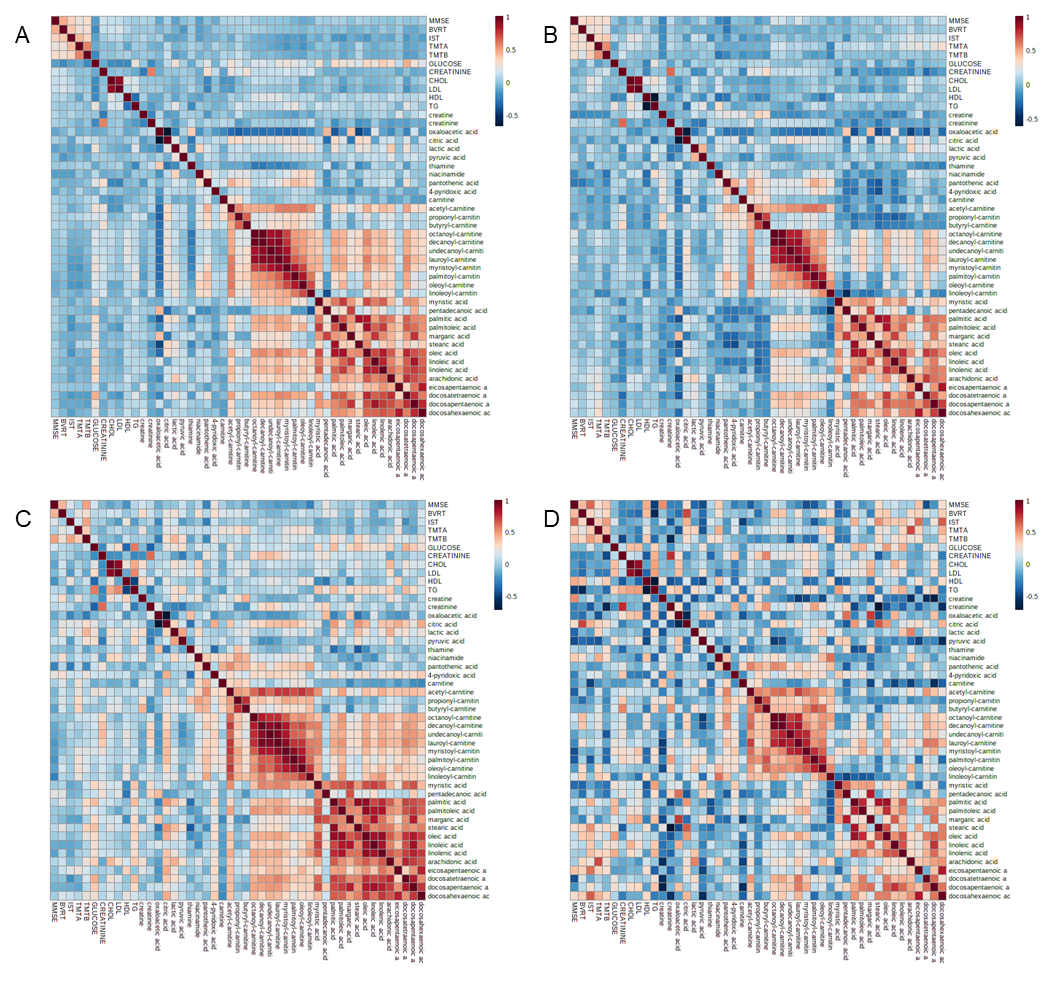


**Figure S1.** Pearson’s correlation analysis between metabolomics, biochemical and neuropsychological variables within the four study groups stratified according to the ApoE-ε4 genotype and sex: female non-carriers (A), male non-carriers (B), female carriers (C), and male carriers (D).
